# Impact of Prevnar vaccination on nasopharyngeal carriage of *Streptococcus pneumoniae* in healthy children in New Caledonia

**DOI:** 10.1186/1753-6561-5-S1-P13

**Published:** 2011-01-10

**Authors:** Julie Scotet, Francine Baumann, Myrielle Dupont-Rouzeyrol, Catherine Grangeon, Shahin Oftadeh, Benoit Garin, Suzanne Chanteau, Simon Le Hello

**Affiliations:** 1 Institut Pasteur de Nouvelle-Calédonie, Nouméa, 98800, New Caledonia; 2 Centre Protection Maternelle et Infantile, Nouméa, 98800, New Caledonia; 3 Pneumococcal Reference Laboratory, Westmead Hospital, Westmead NSW 2145, Australia

## 

We assessed the impact of the heptavalent pneumococcal conjugate vaccine (PCV7) on the nasopharyngeal carriage of *Streptococcus pneumoniae* in healthy children aged 2 to 24 months, four years after its implementation in New Caledonia. The data were compared with those obtained, before the introduction of the vaccine, in the same target population

From February to October 2008, 592 children were enrolled prospectively, regardless of their vaccinal status. Between 2002 and 2008, the prevalence of the overall pneumococcal carriage and of vaccine type carriage decreased significantly, respectively 52.3% to 42.1% (*p < 10^-3^)* and 46.9% to 22.2% *(p < 10^-3^).* This reduction was offset by an increase (20.8% to 29.0%, *p = 0.013*) of the carriage of non-vaccine type pneumococci with reduced susceptibility to penicillin (PRSP), notably the serotypes 15B (*p=0.027*) and 19A (*p=0.001*). This increase in PRSP carriage was marked in the Northern Province (*p = 0.005*) and among Melanesian children (*p = 0.009*). Surprisingly this increase was mainly attributed to the vaccine type 19F (*p < 10^-3^*).

In conclusion, as expected, the PCV7 vaccine led to a decrease of the pneumococcal carriage and the replacement of vaccine strains by non vaccine strains, however increasingly resistant to penicillin. In the Northern Province, the increasing carriage of penicillin resistant 19F strains escaping the vaccine is of concern and justifies a further comprehensive analysis using MLST genotyping.

